# Malignant Trigeminal Nerve Sheath Tumor and Anaplastic Astrocytoma Collision Tumor with High Proliferative Activity and Tumor Suppressor *P*53 Expression

**DOI:** 10.1155/2014/153197

**Published:** 2014-10-15

**Authors:** Maher Kurdi, Hosam Al-Ardati, Saleh S. Baeesa

**Affiliations:** ^1^Department of Pathology, Faculty of Medicine, King Abdulaziz University, Jeddah 21589, Saudi Arabia; ^2^Department of Pathology, King Faisal Specialist Hospital, Jeddah 21499, Saudi Arabia; ^3^Division of Neurosurgery, Faculty of Medicine, King Abdulaziz University, P.O. Box 80215, Jeddah 21589, Saudi Arabia

## Abstract

*Background*. The synchronous development of two primary brain tumors of distinct cell of origin in close proximity or in contact with each other is extremely rare. We present the first case of collision tumor with two histological distinct tumors. *Case Presentation*. A 54-year-old woman presented with progressive atypical left facial pain and numbness for 8 months. MRI of the brain showed left middle cranial fossa heterogeneous mass extending into the infratemporal fossa. At surgery, a distinct but intermingled intra- and extradural tumor was demonstrated which was completely removed through left orbitozygomatic-temporal craniotomy. Histopathological examination showed that the tumor had two distinct components: malignant nerve sheath tumor of the trigeminal nerve and temporal lobe anaplastic astrocytoma. Proliferative activity and expressed tumor protein 53 (TP53) gene mutations were demonstrated in both tumors. *Conclusions*. We describe the first case of malignant trigeminal nerve sheath tumor (MTNST) and anaplastic astrocytoma in collision and discuss the possible hypothesis of this rare occurrence. We propose that MTNST, with TP53 mutation, have participated in the formation of anaplastic astrocytoma, or vice versa.

## 1. Introduction

Collision tumors are defined as two tumors with discrete histology appearing simultaneously and in proximity to each other at the same anatomic location. Such occurrence in the brain is relatively rare and few case reports of primary collision brain tumors were published in the literature, which are summarized in Table 1. The mechanism of this rare entity remains unclear, but some hypotheses for collision tumors were proposed.

Pituitary adenoma and craniopharyngioma in collision is the commonest association, which was recently reviewed by Jin et al., of 14 reported cases [1]. Meningioma is the second frequently reported tumor to collide with another tumor of different cell type, mostly astrocytoma [2–7]. The association of cranial nerve sheath tumor of the trigeminal and acoustic nerves has been reported to collide with epidermoid tumors in the posterior fossa in 3 cases [8–10].

We describe the first report of malignant nerve sheath tumor of the trigeminal nerve in collision with anaplastic astrocytoma of temporal lobe and discuss the possible theory of this rare association.

## 2. Case Presentation

A 54-year-old woman presented with 8-month history of progressive atypical left facial pain and numbness. She had no previous illness, and her elder brother died few years ago from parietal glioblastoma multiforme. General physical examination was unremarkable; there were no cutaneous stigmata of neurofibromatosis. The neurological examination demonstrated facial hypoesthesia in the distribution of the second and third divisions of the trigeminal nerve. The rest of cranial nerves and neurological examination were within normal.

**Figure 1:**
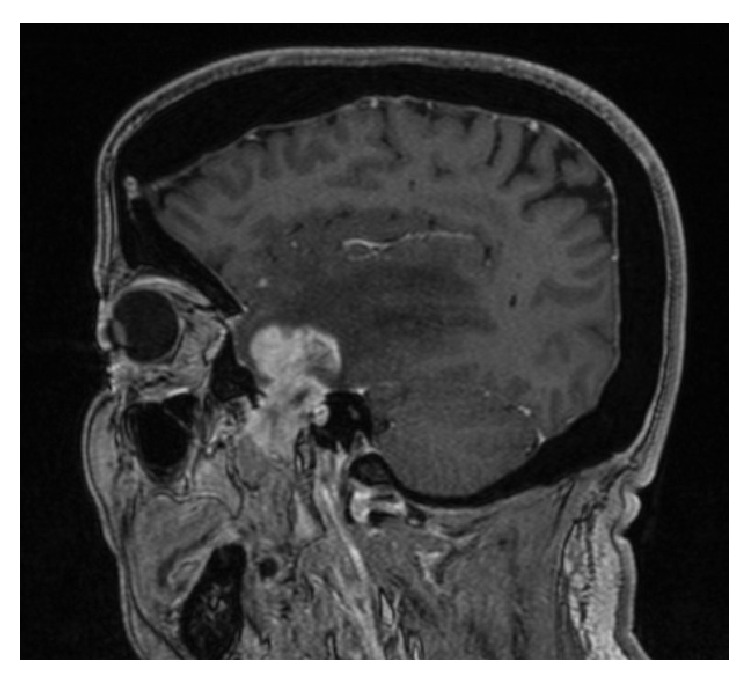
Preoperative enhanced parasagittal T1-WI MRI demonstrates extra-axial heterogeneously dumbbell-shaped enhancing tumor in the left middle fossa extending into infratemporal fossa.

**Figure 2:**
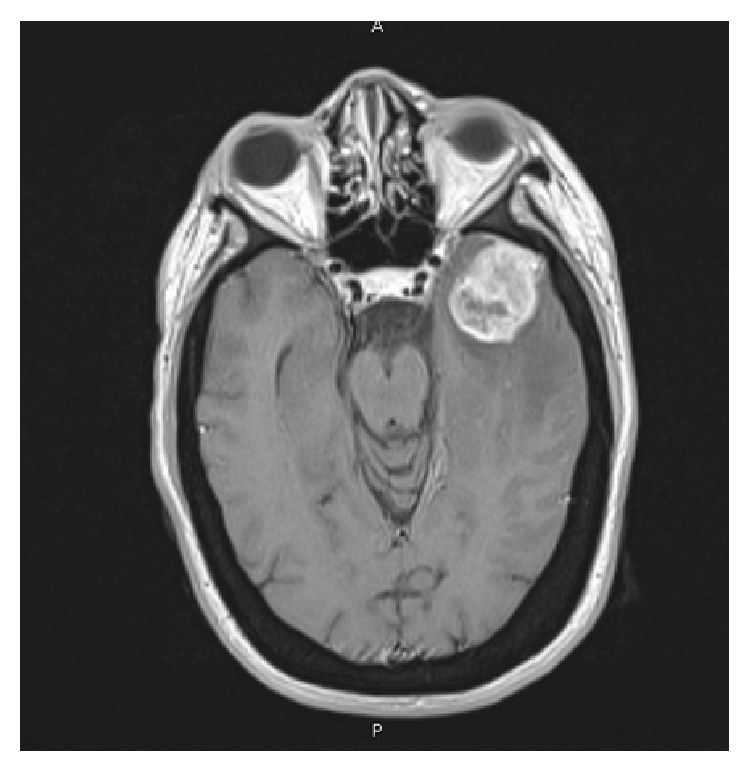
Axial T1-WI MRI demonstrating mass effect and infiltration to the temporal lobe with vasogenic edema.

**Figure 3:**
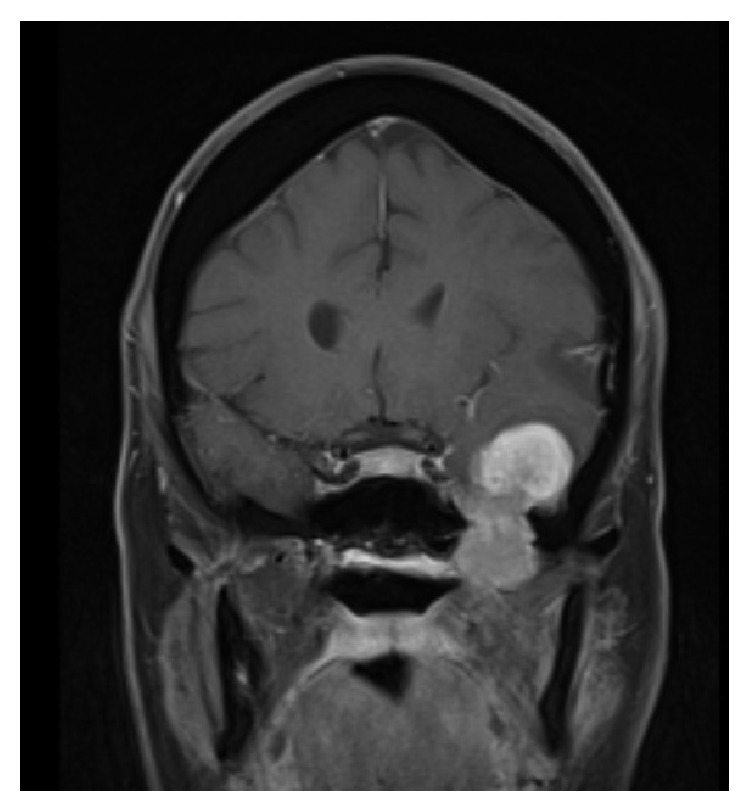
Coronal T1-WI MRI demonstrating middle cranial fossa tumor extending to the infratemporal region.

Magnetic resonance imaging (MRI) of the brain revealed a 40 × 25 × 40 mm isointense lesion that enhanced heterogeneously following intravenous Gadolinium administration enhancing lesion in the anterior part of left middle cranial fossa involving the anterior part of the temporal lobe and extends to the infratemporal fossa through foramen ovale (Figures 1, 2, and 3). The tumor had intra-axial enhanced solid and cystic unenhanced components in the left anterior temporal lobe with surrounded vasogenic edema. The radiological findings were likely consistent with trigeminal schwannoma. Surgery was performed via temporal extradural and intradural approach, with neuronavigation guidance, through left orbitozygomatic-temporal craniotomy. The tumor was seen extradurally easily separable from the dura. It was noticed that it is smaller than the expected size from the MRI. The dura was then opened and a striking intra-axial soft tumor was noted in the superior and middle temporal gyri. Complete microsurgical resection, with ultrasonic aspirator, of the intradural portion of the tumor was performed through anterior temporal lobectomy and the dura was closed. The extradural portion was exposed with drilling of middle fossa floor into the infratemporal fossa and enlarging foramen ovale laterally. The tumor was encapsulated and removed completely, with ultrasonic aspirator, and few of the involved trigeminal nerve fascicles were sectioned.

**Figure 4:**
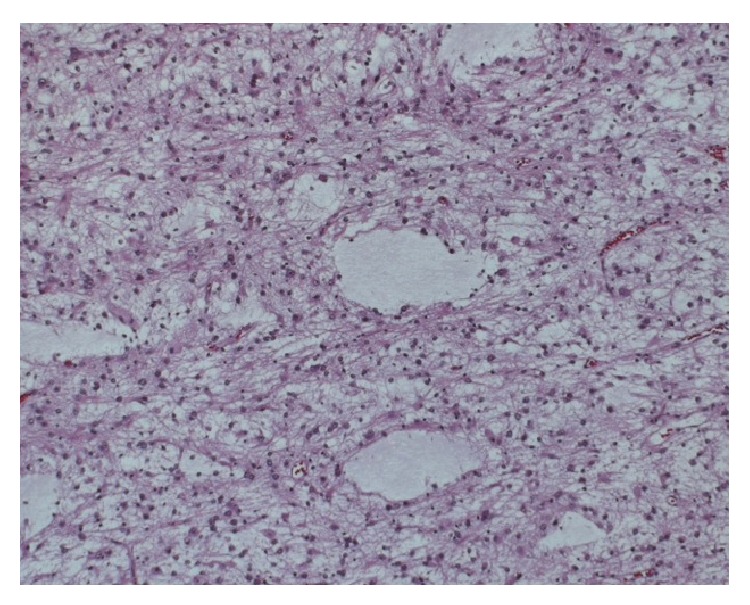
Microphotograph shows anaplastic astrocytoma with numerous mitoses (Hematoxylin & Eosin, original magnification ×20).

**Figure 5:**
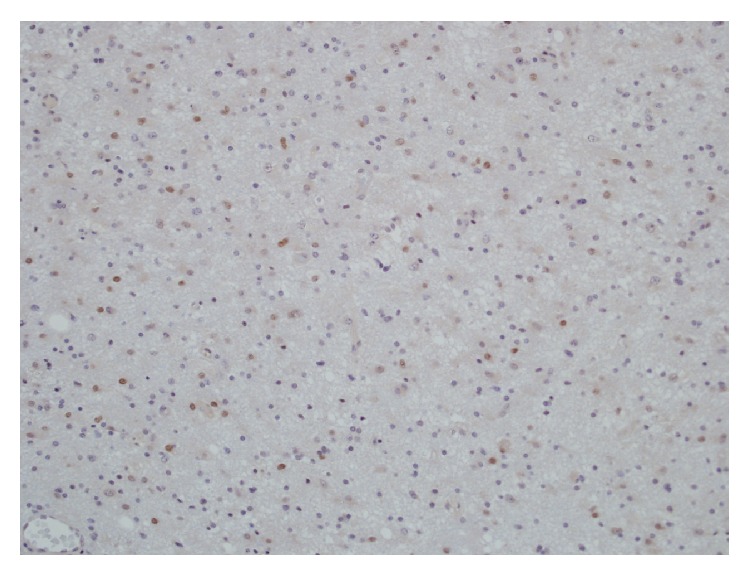
Microphotograph demonstrating significant* P*53 immunopositivity of anaplastic astrocytoma.

**Figure 6:**
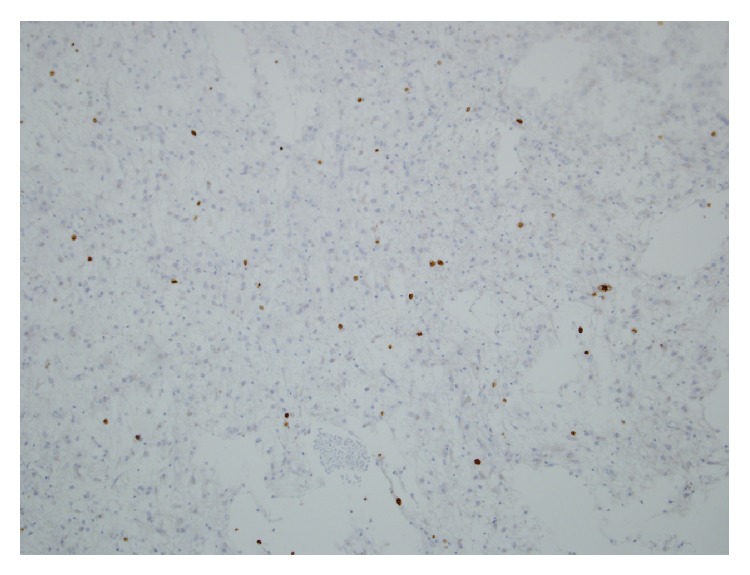
Ki 67 proliferative index of 10% per 10 HPF in the anaplastic astrocytoma.

**Figure 7:**
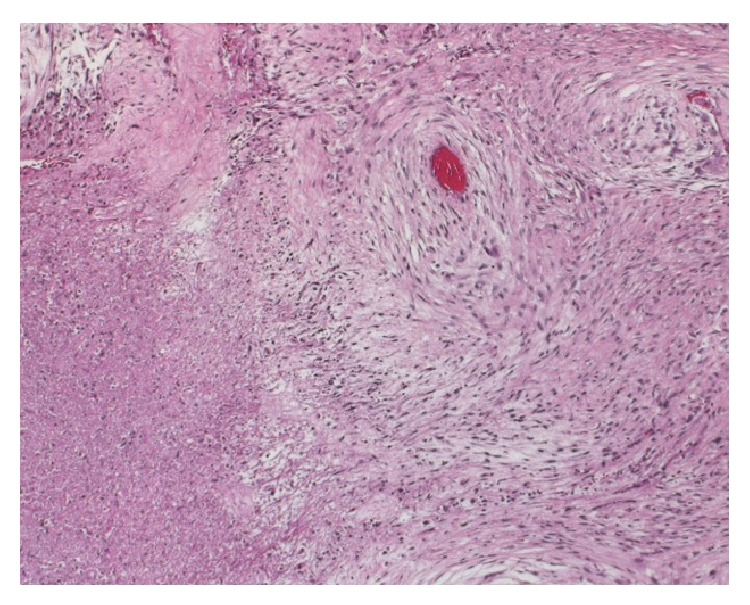
Microphotograph shows MTNST with extensive atypia, mitosis, and necrosis (Hematoxylin & Eosin, original magnification ×20).

**Figure 8:**
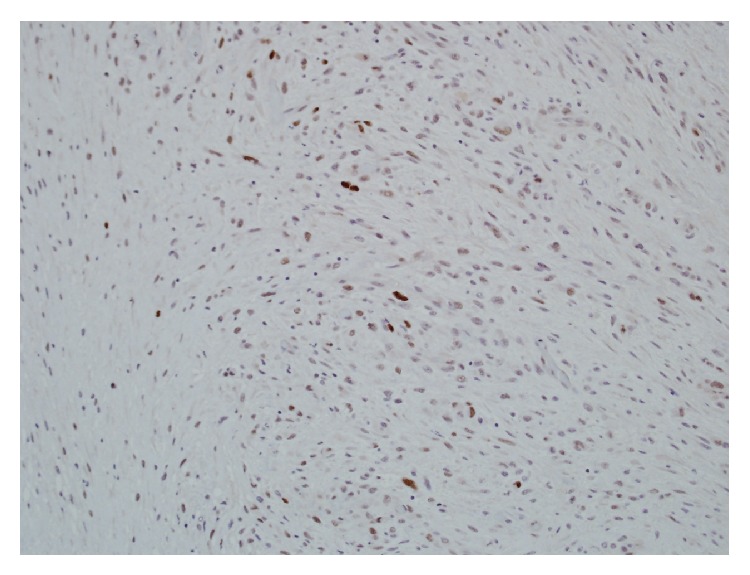
*P*53 immunopositivity of MTNST was apparent.

**Figure 9:**
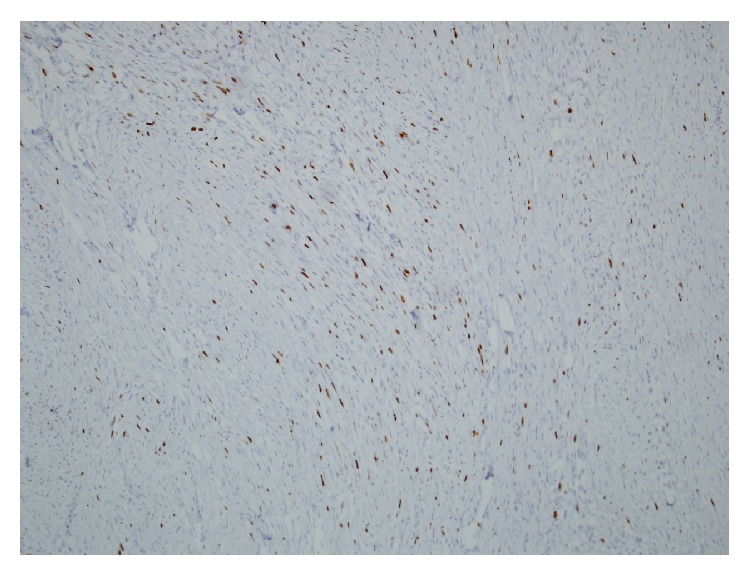
Ki 67 proliferative index of 10% per 10 HPF in MTNST.

Microscopically, the tumor showed two components. The first component of temporal lobe specimen revealed a mitotically active malignant glial neoplasm, with no endothelial proliferations or necrosis (Figure 4). These morphological features were consistent with anaplastic astrocytoma (WHO grade III). Immunohistochemistry study revealed a strong glial fibrillary acidic protein (GFAP) and* P*53 positivity (Figure 5). Ki-67 proliferative index showed a mild to moderate (5–10% per 10 HPF) proliferation (Figure 6). The second component from the extradural specimen revealed a malignant spindle cell neoplasm arranged in a fascicular pattern and embedded in a loosely textured background, with apparent numerous mitosis and tumor necrosis (Figure 7). Immunohistochemically, the spindle cells were strongly positive for vimentin and S-100 proteins, and apparent* P*53 expression (Figure 8). Ki-67 proliferative index showed a mild to moderate (5–10% per 10 HPF) proliferation (Figure 9). The final diagnosis was consistent with two coexisting intermingled anaplastic astrocytomas and malignant trigeminal nerve sheath tumor (MTNST).

**Figure 10:**
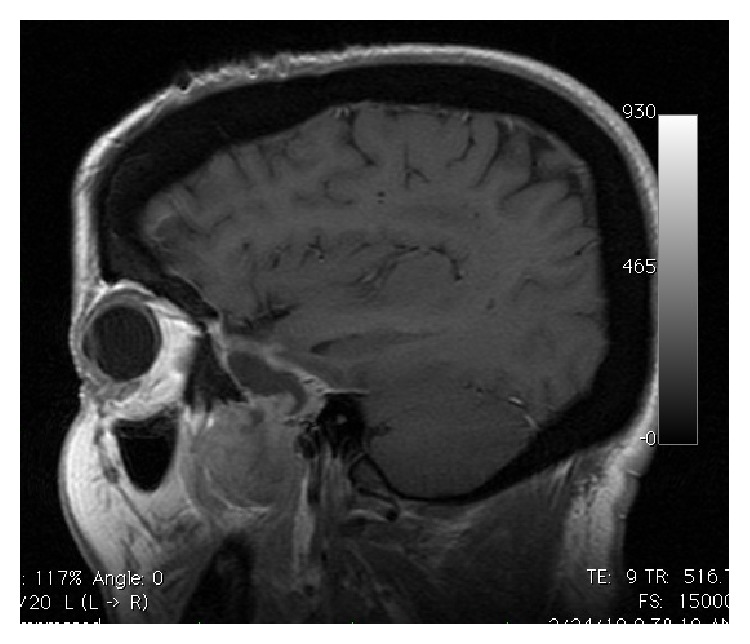
Postoperative (24 hr) follow-up T1-WI parasagittal MRI demonstrates complete resection of tumor from the infratemporal region.

**Figure 11:**
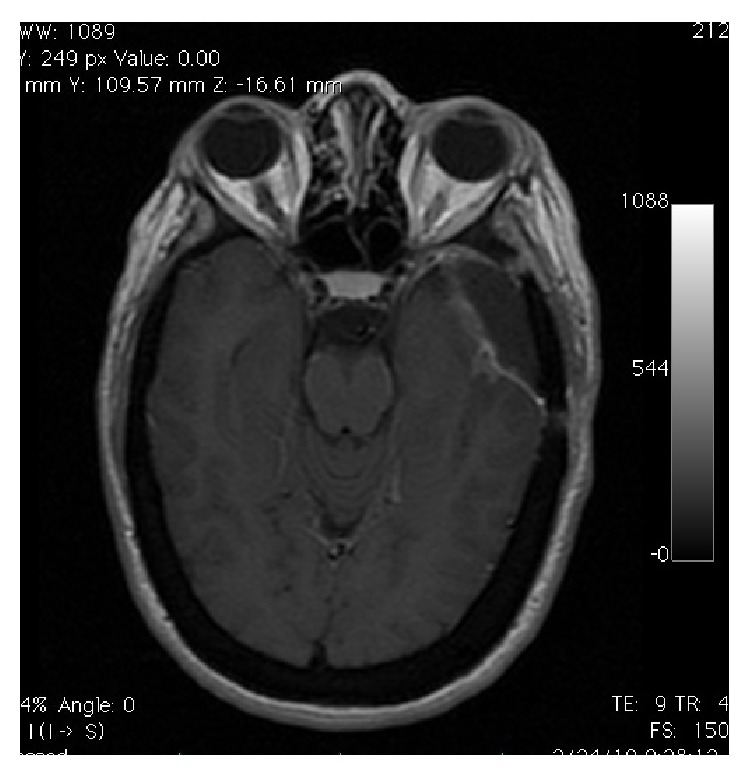
Postoperative (24 hr) follow-up T1-WI axial MRI demonstrates complete resection of tumor from the middle region.

**Figure 12:**
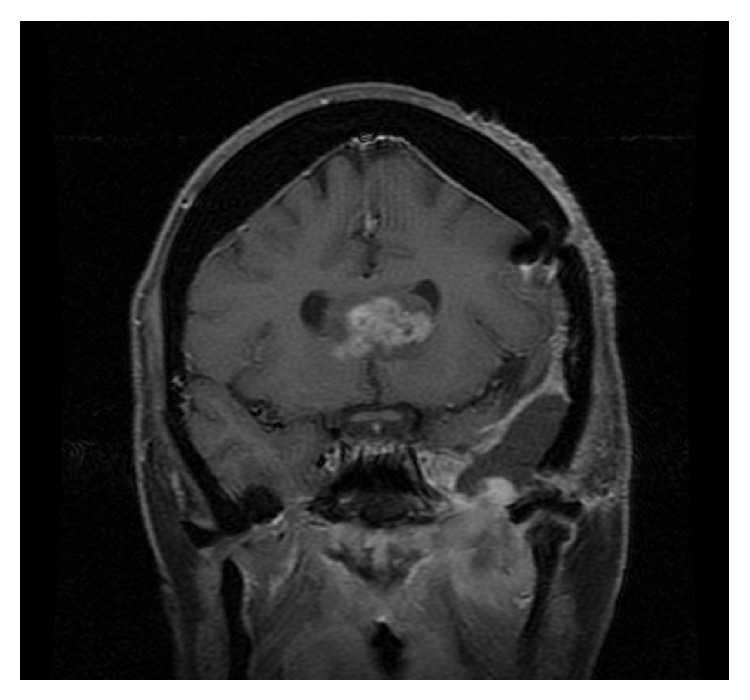
Postoperative (8-month) follow-up T1-WI axial MRI demonstrates recurrent local tumor, likely from anaplastic astrocytoma component, and distant spread to the anterior part of corpus callosum.

The patient had uneventful postoperative period, with significant improvement of facial pain. Postoperative brain MRI scans demonstrated a complete resection of both tumors (Figures 10 and 11). After counseling, the patient decided not to receive any further adjuvant therapy. She was readmitted 8 months later for palliative care due to progressive cognitive function decline. Brain MRI scans revealed left temporal leptomeningeal enhancement at resection cavity at the temporal and infratemporal regions, as well as new heterogeneous enhancing lesion at anterior part of corpus callosum; the latter is likely from the anaplastic astrocytoma component (Figure 12). She passed away 9 months from surgery from tumor progression causing raised intracranial pressure and brain herniation.

## 3. Discussion

Collision tumor is described as two tumors with discrete histology occurring simultaneously and in proximity to each other at the same location. Such intracranial occurrence is rare with few reported cases in the literature (Table 1). The mechanism of this entity remains unclear, but some hypotheses were proposed. The observation that a significant number of the reported cases had their tumor localization in juxtaposition raises the possibility that one tumor may act as an irritating agent for the local proliferation and growth of the other [11]. Surgical trauma, ionizing radiation, and genetic factors may influence tumor development [4]. This theory was suggested in cases where the tumors are adjacent to each other. However, it may not explain why this collision occurred in our rare case making the pathogenesis more complex and multifactorial. Most of reported cases in the literature had reported collision of gliomas and meningiomas [2–7]. Glioma may develop due to neoplastic transformation of the reactive glial cells surrounding a meningioma [5]. This process may be mediated by locally acting oncogenic factors. The most suspected substance is platelet-derived growth factor subunit alpha-R (PDGF-alpha-R), which is the main receptor in astrocytoma [12]. Astrocytoma growth is probably stimulated by PDGF in an autocrine mechanism. It is possible to develop a meningioma as secondary malignant neoplasm due to transformation of the arachnoid cells in response to the growth of a subjacent glioma or after radiation therapy [4].

In our case, we present two different tumors: MTNST and anaplastic astrocytoma presented as collision. MPNSTs are rare malignancies with a reported frequency in the general population of 0.001% [13]. They arise in two principal forms, sporadic (50–70%) and in association with neurofibromatosis type 1 (30–50%) [14]. Unlike other MTNSTs, those involving the trigeminal nerve are not often associated with neurofibromatosis and seem to arise de novo or, rarely, through malignant transformation of preexisting benign Schwannoma [15].

Trigeminal nerve tumors can occur in the posterior fossa, the middle fossa, or extend into multiple cranial fossae. There are 20 cases of MTNSTs reported in literatures since 1978 in which one of them was associated with neurofibromatosis while some cases had extracranial component, and none of them was associated with another different brain tumor [13–18]. Our case is unusual; the patient had MTNST with no history of neurofibromatosis, tumor extended extracranially into the infratemporal region through foramen ovale, and it was intermingled with anaplastic astrocytoma of temporal lobe, which has not, to our knowledge, has been reported as a collision tumor.

Schwannoma classically appears on MRI hypointense in T1 images and hyperintense on T2 images, with marked enhancement after gadolinium administration. The schwannoma in our patient was described as an extra-axial lesion in the left middle temporal fossa, continuous in a dumbbell-shaped fashion to infratemporal region. It was hypointense in T1- and hyperintense in T2- weighted images and enhanced significantly after Gadolinium administration. There was an intermingled cystic component in the left anterior temporal lobe region with surrounded vasogenic edema intermingled with the tumor.

Malignant peripheral nerve sheath tumors (MPNST) histologically consists of a mixture of plump spindle cells with large hyperchromatic pleomorphic nuclei [19]. Numerous mitotic figures, necrosis and epineural invasion, may help to confirm the diagnosis. Our patient showed identical histopathological features of MPNST in the first tumour specimen. We performed immunohistochemical studies for* P*53, which revealed marked expression in more than 50% of tumor cells of both malignant neoplasms in our case. Tumor suppressor* P*53 is a protein, encoded by TP53 gene, is crucial in multicellular organisms, where it regulates the cell cycle, and, thus, functions as a tumor suppressor, inducing apoptosis and preventing cancer. When* P*53 immunostain is positive, it means that the tumour may have TP53 gene mutation. This component might be derived from a single precursor cell that undergoes divergent differentiation in the evolution of the tumor. However, the observed overexpression of the gene product, possibly the reflection of the TP53 gene mutation, suggests a role for a* P*53 in the proliferation of glial cells from anaplastic glioma and vice versa. There was one reported case in the literature of a* P*53 expression in MPNST and neurofibroma in which the possible role of the* P*53 in the progression of neurofibroma to MPNST was suggested [20].

Total tumor excision is the preferred treatment option in these cases, and radiosurgery is usually given as adjuvant therapy for malignant or residual or recurrent benign tumors [19]. MTNST carries a poor prognosis having a local recurrence rate of 54% and a 5-year survival rate of 34% [21]. Ki 67 proliferative index is a strong predictor factor in MTNST. Moderate to high Ki 67 proliferative index predicts a reduced survival rate. Our case showed mild to moderate proliferative activity of 10% in both tumors.

The patient had an uneventful recovery of her symptoms within 2 months from surgery. She refused to receive any adjuvant therapy due to her brother's death from glioblastoma. She presented with acute confusion, 9 months after surgery, and MRI showed local recurrence from both tumors and distant recurrence at the corpus callosum, which was believed from the anaplastic astrocytoma. She died from progressive raised intracranial pressure due to severe tumoral edema. Autopsy examination was not performed in respect to family decision.

## 4. Conclusion

We herein for the first time report MTNST and anaplastic astrocytoma in collision with similar proliferative activity and TP53 mutation which may have induced the malignant features of the first and may have played part of the proliferation of the latter, or vice versa.

## Figures and Tables

**Table 1:** Reported primary brain collision tumors in the literature.

Author (year) reference	Histological type of collision tumor
Tajika et al. (1989) [11]	Intrasellar gangliocytoma and pituitary adenoma

Kitaoka et al. (1986) [8]	Posterior fossa epidermoid tumor and trigeminal neuroma

Vaquero et al. (1990) [2] Dario et al. (1995) [3] Hakan et al. (1998) [4] Mitsos et al. (2009) [6] Prayson et al. (2002) [5] Khalatbari et al. (2011) [7]	Meningioma and astrocytoma (including glioblastoma)

Suh et al. (2003) [22]	Suprasellar chordoid glioma and Rathke's cleft cyst

Johnson et al. (2001) [23]	Oligodendroglioma and ganglioglioma

Perry et al. (2001) [24]	Oligodendroglioma and pleomorphic xanthoastrocytoma

Muzumdar et al. (2001) [25]	Pontine glioma and epidermoid tumor

Vajtai et al. (1997) [26] Evans et al. (2000) [27]	Pleomorphic xanthoastrocytoma and ganglioglioma

Goodman et al. (1991) [9] Kleinpeter and Koos (1994) [10]	Acoustic schwannoma and epidermoid cyst

Basil et al. (2011) [28]	Primary CNS B-cell lymphoma and anaplastic astrocytoma

Jin et al. (2013) [1] (review of 14 cases)	Pituitary adenoma and craniopharyngioma

Woo et al. (2014) [29]	Central neurocytoma and epidermoid tumor

Current case	Trigeminal nerve sheath tumor and anaplastic astrocytoma
